# Gut Microbiome Composition Abnormalities Determined Using High-Throughput Sequencing in Children With Tic Disorder

**DOI:** 10.3389/fped.2022.831944

**Published:** 2022-05-04

**Authors:** Yanping Wang, Houxi Xu, Miao Jing, Xiaoyue Hu, Jianbiao Wang, Ying Hua

**Affiliations:** ^1^Department of Neurology, The Affiliated Wuxi Children's Hospital of Nanjing Medical University, Wuxi, China; ^2^Key Laboratory of Acupuncture and Medicine Research of Ministry of Education, Nanjing University of Chinese Medicine, Nanjing, China

**Keywords:** tic disorder, gut microbiota, high-throughput sequencing, 16S rRNA, abnormalities

## Abstract

**Object:**

To investigate the distribution characteristics of gut microbiota in children with tic disorder (TD) and the possible role of these characteristics in the pathogenesis of TD.

**Methods:**

The medical records of 28 children with TD treated at Wuxi Children's Hospital from January 1 to October 31, 2020, and 21 age-matched healthy children (controls) were included. The relative quantification of bacterial taxa was performed using 16S ribosomal RNA gene amplicon sequencing.

**Results:**

There was no significant difference in the alpha diversity of gut microbiota between the TD and control groups. Analyses of beta diversity were able to differentiate the TD patients from the healthy controls based on their gut microbiota. At the phylum level, the two groups were mainly composed of four phyla, Firmicutes, Actinobacteria, Bacteroidetes, and Proteobacteria. There were significant differences in Firmicutes and Actinobacteria between the two groups (*P* <0.05). At the level of genera, the abundance of *Bifidobacterium* and *Collinsella* reduced while that of Ruminococcaceae unclassified, *Prevotella, Faecalibacterium, Coprobacillus*, and *Odoribacter* increased in the TD group compared to that in the control group. The intergroup differences were significant (*P* < 0.05).

**Conclusion:**

The abnormal composition of gut microbiota in children with TD suggests that the change in gut microbiota may play an important role in TD development.

## Introduction

Tic disorder (TD) is a childhood-onset neuropsychiatric and neurodevelopmental disorder (1, 2). Its main manifestations are involuntary, repetitive, rapid, purposeless, motor tics and/or vocal tics of one or more muscles. The age of onset of TD is 2–21 years, with TD most commonly developing between the ages of 5 and 10 years (3). TDs are more common among male patients than among female patients, with the male to female ration being 3–5:1. In recent years, the incidence of TD has increased (4). Currently, the incidence of TD in Chinese children is ~6.1% (5). However, the etiology and pathogenesis of TD have not yet been fully explored. Most scholars believe that this disease may be the result of interactions among genetic factors, environmental factors, and neurotransmitters during the growth and development of children (6, 7).

Studies have shown that the gut microbiota is closely related to central nervous system diseases, such as epilepsy, autism spectrum disorder (ASD), and autism, attention deficit hyperactivity disorder (ADHD) (8–11). ADHD is the most common comorbidity of TD, and the two conditions share similar etiological characteristics and pathogenesis (12). Several previous studies have shown that the composition of the gut microbiota in children with ADHD was significantly different from that in healthy children (9, 10, 13). The gut is called the “second brain” or “gut-brain” in humans (14). The gut and brain interact through the bidirectional pathway of the brain-gut axis, which affects the central nervous system. The gut microbiota is the core of the microbiota-gut-brain axis, as an important mediator for the mutual adjustment of the brain and the gastrointestinal tract. It not only regulates the body's physiological functions but also changes the brain development trajectory of humans and animals, thereby regulating the behavior and cognitive functions of the host (15). Microbiota-generated metabolites, especially the neurotransmitters such as γ-aminobutyric acid (GABA), glutamate and histamine, could affect brain activity in the microbiota–gut–brain bidirectional communication (16). Therefore, an abnormal composition of the gut microbiota may lead to abnormal neurotransmitter secretion, which could promote the development of neuropsychiatric diseases. Zhao et al. reported that severe TD in a child was markedly ameliorated after fecal microbiota transplantation, promoting the consideration of the possible association between gut microbiota and TD development (17). We analyze the microecological distribution of the gut microbiota in children with TD and healthy controls by high-throughput sequencing methods.

## Materials and Methods

### Subjects

Twenty-eight children with TD who visited the pediatric clinic of Wuxi Children's Hospital from January 2020 to October 2020 were selected as the research objects. The patients were aged 6–14 years, with the average age being 8.2 ± 1.2 years. Seventeen of the children were male and the rest were female; the disease duration in these children ranged from 6 months to 5 years. The criteria for TD patients consisted of the following: (1) diagnosed as TD through a comprehensive assessment according to the Expert Consensus on the Diagnosis and Treatment of Tic Disorders in Children (2017 Practical Edition) (18) and the 5th edition of the Diagnostic and Statistical Manual of Mental Disorders (DSM-5) (19); (2) have never taken any medications to treat their TD before enrollment. The exclusion criteria were as follows: (1) a history of intellectual disability, autism, mood disorders, or other neuropsychiatric disorders; (2) presence of chorea, epilepsy, and other extravertebral diseases such as hepatolenticular degeneration, Parkinson's disease and athetosis; (3) a history of conditions such as obesity, precocious puberty, asthma, heart disease, gastrointestinal disease, and reproductive system defects; (4) use of systemic or local glucocorticoids, immunosuppressants, and antihistamines within 15 days before study enrollment; (5) presence of other co-morbidities related to Tourette syndromes such as ADHD or obsessive-compulsive disorder (OCD) and anxiety disorders; and (6) presence of other serious illnesses. Two senior neurological clinicians jointly assessed and completed the inclusion and exclusion of samples. Twenty-one children, 13 males and eight females, without TD who underwent health checkups in our hospital during the same period were included as the control group. They had no known physical illnesses or any of the aforementioned major neuropsychiatric diseases. The healthy children were aged 5–14 years, with an average age of 7.9 ± 2.0 years. The characteristics of the subjects are shown in Table 1. Both the control and TD groups did not receive antibiotics and probiotics within 2 months before enrollment. This study was approved by the ethics committee of Wuxi Children's Hospital (approval number: WXCH2019-08-006). All the enrolled participants and their family members signed an informed consent form.

**Table 1:** Characteristics of patients with TD and healthy control children.

	**TD (*n* = 28)**	**Controls (*n* = 21)**	***P-*** **values**
Sex (*n*, %)			0.585
Boy	17 (60.7)	13 (61.9)	
Girl	11 (39.3)	8 (38.1)	
Age (mean ± SD)	8.2 ± 1.9	7.9 ± 2.1	0.569
Age, range	6–14	5–14	
BMI	19.3 ± 1.9	18.8 ± 1.7	0.248

### Methods

#### Sampling

Design of the *Record Form for Children with Tic Disorder* for collection of clinical data.

The form was designed to gather data on the child's general condition and date of TD onset, date of the first hospital visit for TD, first symptoms, current symptoms (specific symptoms of motor and vocal tics in children with TD), symptom frequency, daily activities, and learning and social situations. For each child enrolled in the TD group, 100 mg of fecal sample was collected in three sets of 2-mL sterile centrifuge tubes. The tubes were numbered according to the order of entry. Samples in two of the tubes were used for DNA extraction, and the remaining tube was reserved. All fecal samples were processed within 30 min after collection and then stored in a refrigerator maintained at −80°C.

#### DNA Extraction, Library Construction, and Sequencing

The E.Z.N.A.^®^ Soil DNA Kit (Omega Biotek) was used to extract DNA from samples in accordance with the operating instructions. After DNA extraction, the V3-V4 region of 16s rDNA was amplified by PCR. The PCR product was purified by 2% agarose gel electrophoresis, and the target fragment was cut and recovered. Qubit fluorometer was used to determine the DNA mass concentration of the library, and the KAPA Library Quantification Kit was used to quantitatively determine the molar concentration of the library DNA. After the library was mixed and denatured, the amplified products were subjected to paired-end sequencing on the Illumina Novaseq sequencing platform.

#### Bioinformatics Analysis

Thecutadapt software was used to filter sequencing data to obtain high-quality clean data. The search software was used for sequence analysis and to classify sequences with a similarity of ≥97% as the same operational taxonomic units (OTUs). To obtain the species classification information corresponding to each OTU, sequences were compared with those in the Silva (SSU128) 16S rRNA database (http://www.arb-silva.de) to obtain the phylum to genus information for each OTU. The relevant analyses of the gut microbiota, including species annotation and evaluation, alpha diversity, beta diversity, and species difference analyses, were conducted using the I-Sanger cloud analysis platform (http://www.i-sanger.com/) of Meiji Biotechnology. Alpha diversity analysis is the analysis of species diversity in a single sample, which can reflect the richness and diversity of the microbial community. The commonly used metrics are the Shannon, Simpson, ACE, and Chao indexes, among which the ACE and Chao1 indexes reflect community richness and the Shannon and Simpson indexes reflect community diversity. Beta diversity analysis is a comparative analysis of the microbial community composition of different samples; it is used to evaluate differences between microbial communities. The commonly used analysis methods include principal component analysis, principal coordinate analysis (PCoA), unweighted pair group method with arithmetic mean analysis (UPGMA), and analysis of similarities (ANOSIM) (20).The Wilcoxon rank-sum test was used to analyze differences in the flora between children with TD and healthy children. *P* < 0.05 was considered statistically significant.

## Results

### Characteristics of the Patients

In all, 28 children with TD were enrolled, including 17 males and 11 females. The patients were aged 6–14 years, with an average age of 8.2 ± 1.9 years. The disease duration range was 6 months to 5 years. The control group comprised 21 children, including 13 males and eight females. The controls were aged 5–14 years, with the average age being 7.9 ± 2.1 years. There were no statistically significant differences in sex and age between the two groups (*P* > 0.05).

### Alpha Diversity Analysis

**Figure 1:**
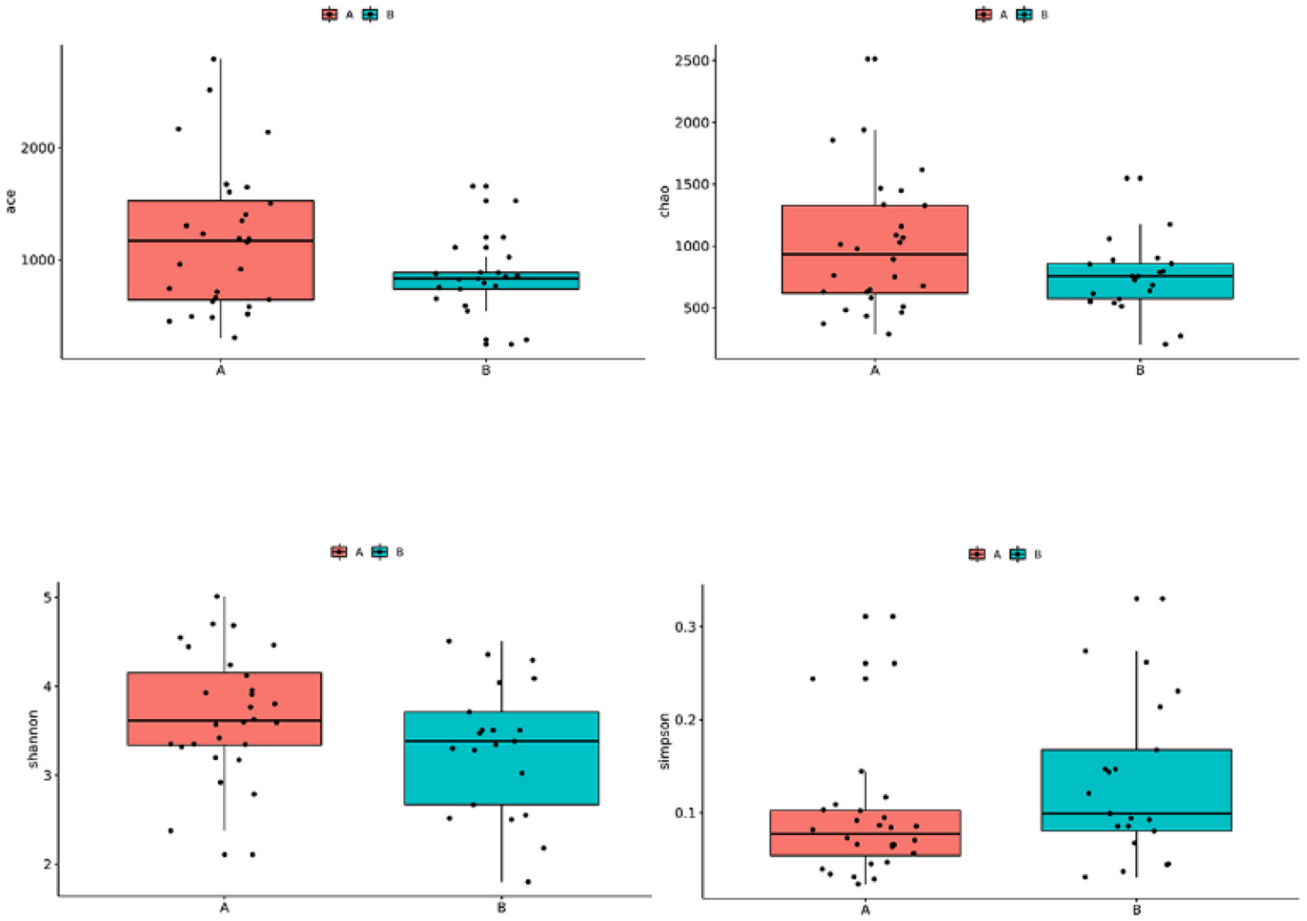
Comparison of the alpha diversity of gut microbiota in children with TD and control children. Group A: TD group; Group B: control group.

The Wilcoxon rank-sum test used to compare the TD and control groups showed no significant difference in the alpha diversity index (Shannon index, Simpson index, ACE index, Chao index) between the two groups (*P* > 0.05) (Figure 1).

### Beta Diversity Analysis

**Figure 2:**
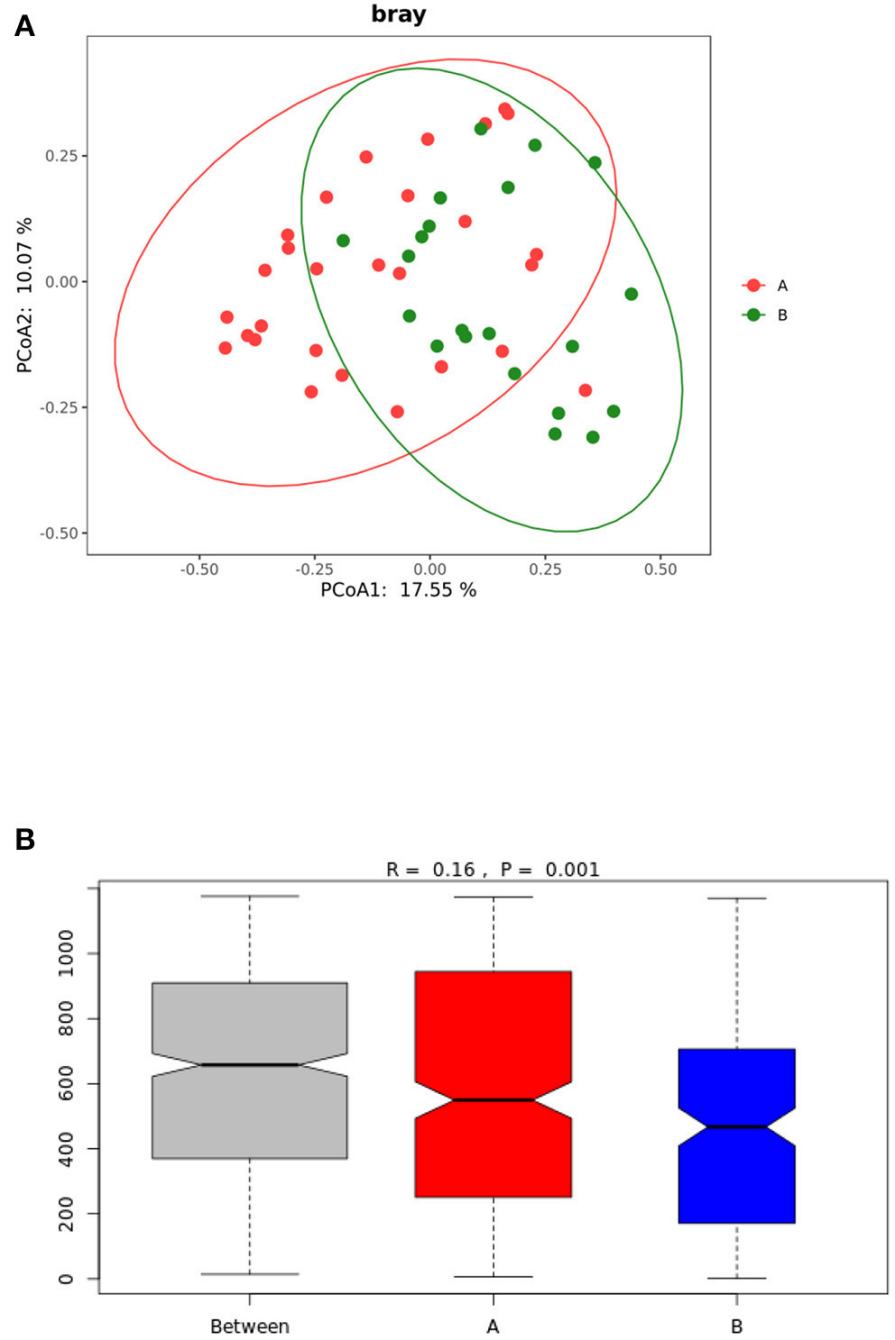
**(A)** PCoA analysis of gut microbiota in TD group and control group. **(B)** ANOSIM analysis of gut microbiota in the TD and control groups. Group A: TD group; Group B: control group.

The beta diversity analysis (PCoA and ANOSIM) (Figure 2) showed significant differences between the gut microbiota of the TD and control groups (*P* < 0.05).

### Comparison of the Bacterial Composition and Structure

#### Differences at the Phylum Level

**Figure 3:**
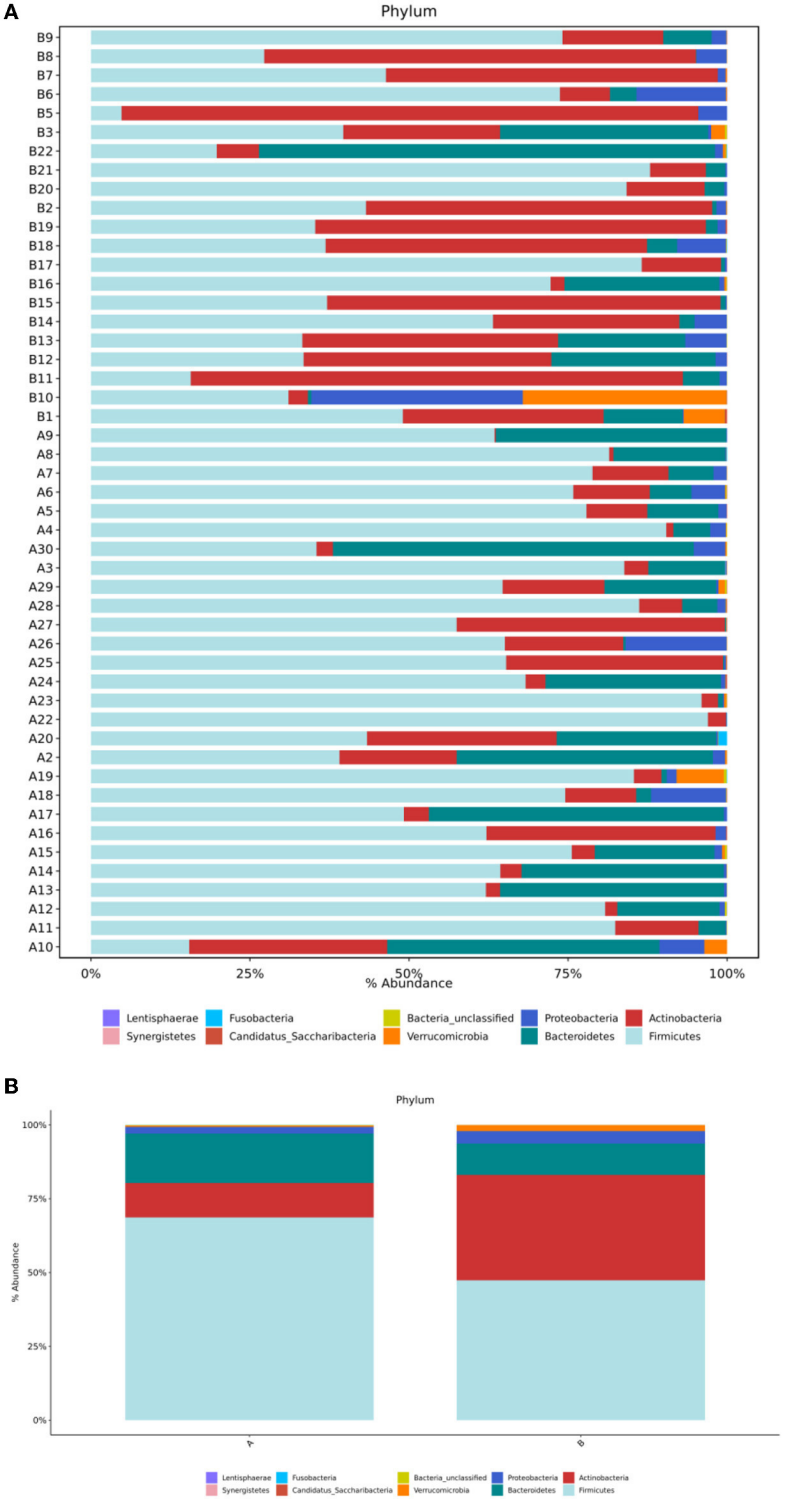
**(A)** Structural composition analysis of the gut microbiota in each sample at the phylum level. **(B)** Comparison of relative abundance and composition of bacteria in the two groups at the phylum level. Group A, TD group; Group B, control group.

Sequence analysis of the TD and the control groups showed that at the phylum level, the gut microbiota of the two groups belonged to 36 phyla, of which 35 phyla were in group A and 31 were in group B. Both groups were mainly composed of four phyla: Firmicutes, Actinobacteria, Bacteroides, and Proteobacteria (Figure 3A). The order of abundance in the TD group was as follows: Firmicutes, 68.64%; Bacteroidetes, 16.74%; Actinobacteria, 11.68%; Proteobacteria, 2.26%; and others, 0.68%. In contrast, the order of abundance in the control group was as follows: Firmicutes, 47.37%; Actinobacteria, 35.7%; Bacteroidetes 10.58%; Proteobacteria, 4.25%; and others, 2.11% (Figure 3B). The Wilcoxon rank-sum test was used to analyze the differences in species abundance between the two groups, and the results showed statistically significant differences in the abundances of Firmicutes (*P* = 0.004) and Actinobacteria (*P* = 0.003) between the TD and control groups.

#### Differences at the Genus Level

**Figure 4:**
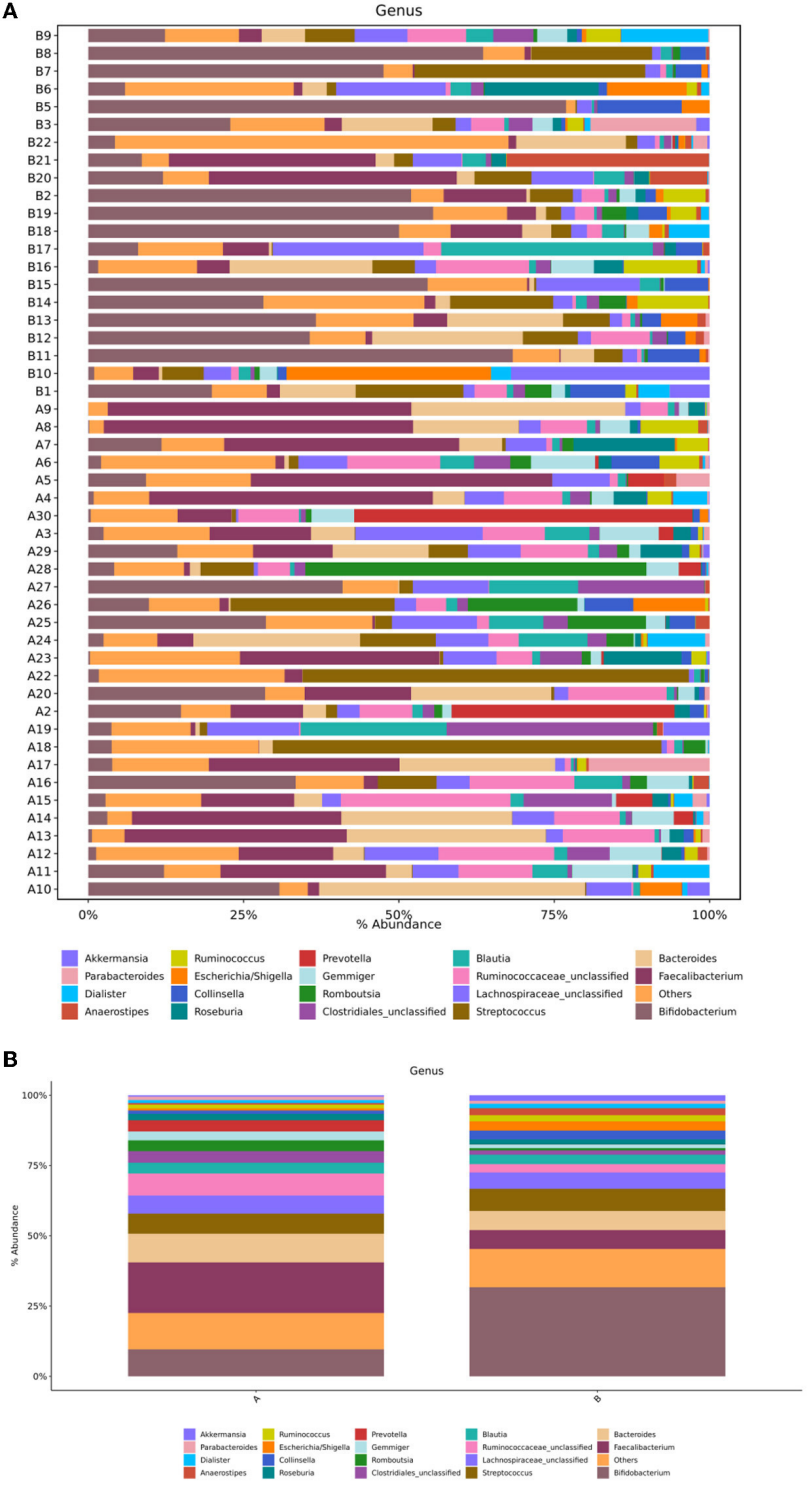
**(A)** Structural composition analysis of the gut microbiota in each sample at the genus level. **(B)** Comparison of relative abundance and composition of bacteria in the two groups at the genus level. Group A, TD group; Group B, control group.

Sequence analysis was performed on children in the TD and control groups. At the genus level, the gut microbiota of the two groups belonged to a total of 167 genera, of which 159 genera were in group A and 140 were in group B. The differences between the samples were large, and the dominant bacteria were different between the two groups (Figure 4A). Figure 4B shows that the 10 most abundant genera in the gut microbiota of the TD group were *Faecalibacterium* (18%), *Bacteroides* (10.21%), *Bifidobacterium* (9.59%), Ruminococcaceae_unclassified (7.8%), *Streptococcus* (7.16%), Lachnospiraceae_unclassified (6.45%), Clostridiales_unclassified (4.07%), *Prevotella* (3.99%), *Romboutsia* (3.88%), and *Blautia* (3.87%). In contrast, the 10 most abundant genera in the gut microbiota of the control group were *Bifidobacterium* (31.69%), *Streptococcus* (7.87%), *Bacteroides* (6.84%), *Faecalibacterium* (6.69%), Lachnospiraceae_unclassified (5.78%), *Blautia* (3.33%), *Escherichia*/*Shigella* (3.23%), *Collinsella* (3.19%), Ruminococcaceae_unclassified (3.02%), and *Anaerostipes* (2.45%). In comparison with the control group, the TD group showed a significantly reduced abundance of *Bifidobacterium* (*P* = 0.001) and *Collinsella* (*P* = 0.03) and significantly increased abundance of Ruminococcaceae_unclassified (*P* = 0.002), *Faecalibacterium* (*P* = 0.006), *Prevotella* (*P* = 0.002*), Gemmiger* (*P* = 0.022), and *Odoribacter* (*P* = 0.014).

## Discussion

TD is a neuropsychiatric disease, and its pathogenesis has not yet been fully explored. Evidence shows that gut microbiota can affect the development of the nervous system and may even cause or aggravate neurological diseases (21). In this study, the results of the α-diversity analysis of the gut microbiota in the TD group and the control group showed that the ace index, Shannon index, Simpson index, and Chaol index were not significantly different between the groups (*P* > 0.05). However, the beta diversity analysis showed that the gut microbial community of children with TD was significantly different from that of healthy children. In the analysis of flora species composition, the two groups showed differences in flora composition and abundance at the phylum and genus levels. In comparison with the control children, the TD group showed a significantly reduced abundance of *Bifidobacterium* and *Collinsella* and significantly increased abundance of*Ruminococcaceae*_unclassified, *Faecalibacterium, Prevotella, Gemmiger*, and *Odoribacter*. As an important probiotic in the intestine, *Bifidobacterium* performs the functions of resisting harmful bacteria and regulating nutrition and the immune response and plays an important role in maintaining the intestinal microecological balance. A reduction in its content can activate the immune system in the intestine, leading to the occurrence of various diseases such as allergic diseases. Some studies in children suggest possible relationships between TS and allergic diseases, such that more research is warranted to clarify the specific nature of these relationships (e.g., longitudinal relationship between variables, whether it is correlational, causal). Given that the neural basis of TDs are relatively understood, it will be important to understand whether and precisely how gut microbiota might impact relevant circuitry, as well as whether altered microbiota precede or are followed by the emergence of TD symptoms (22–24). The gut microbiota of allergic and non-allergic infants shows significant differences during the first year of life (25, 26). In comparison with normal infants and young children, the intestinal tract of allergic infants shows a reduced abundance of the beneficial bacteria *Bifidobacterium* and *Lactobacillus* and increased colonization of *Enterobacter* and *Staphylococcus*. *Lactobacillus* and *Bifidobacterium* have been shown to produce γ-aminobutyric acid (GABA), the primary inhibitory neurotransmitter (27). Reduced GABA concentration in the primary sensorimotor cortex has been suggested to contribute to both motor tics and sensory impairments in TD (28).The low abundance of *Bifidobacterium* can be speculated to cause allergies and affect the release of neurotransmitters in the gut-brain axis, impacting risk for developing TD. It is also possible that the presence of TD is a risk factor for altered gut microbiota through mechanisms not yet understood (e.g., children with TD often have early sensory intolerances which may limit diet, or children with TD may have been more frequently exposed to medications or other illnesses that alter gut microbiota). In recent years, the study of gut microbiota provides a new theoretical basis for probiotics in the treatment of nervous system diseases (29, 30). An increasing number of studies have shown that supplementation of probiotics can improve gut microbiota dysbiosis and play an important role in the treatment of allergic diseases and nervous system diseases (29–32). Limited studies suggest that probiotics may be associated with changes in cognitive function, which can reduce the risk of developing ADHD or ASD (30).This study provides a theoretical basis for the future use of *Bifidobacterium* to treat mild to moderate TD. Moreover, the abundance of *Collinsella* was shown to be reduced in children with TD, and *Collinsella* mainly produces some gas in the intestine, which is believed to be related to abnormal lipid metabolism. However, the relationship between TD and lipid metabolism has not been reported to date, and it needs to be further studied.

This study found an increased abundance of *Prevotella* in children with TD. *Prevotella* is closely related to irritable bowel syndrome (IBS-D), inflammatory bowel disease and other intestinal diseases (33). It contains enzymes that play an important role in the degradation of mucin, which may lead to an increase in intestinal permeability. *Prevotella* has also been confirmed to show a pro-inflammatory effect (34), and its increased expression level may lead to increased expression of inflammatory factors; the levels of inflammatory factors were also shown to be increased in children with TD (35). These inflammatory factors can pass through the blood–brain barrier to affect the development of the nervous system (36). Thus, an increased abundance of *Prevotella* may cause changes in the levels of inflammatory factors, which may also be involved in the pathogenesis of TD. This study also found an increased abundance of *Odoribacter* in children with TD. *Odoribacter* has been also shown to be closely related to neuropsychiatric diseases. In comparison with healthy children, children with pediatric acute onset neuropsychiatric syndrome (PANS) and pediatric autoimmune neuropsychiatric disorders associated with streptococcal infections (PANDAS) show a significantly higher abundance of Odoribacter. The concept of PANS is relatively recent and is derived from research on PANDAS; PANDAS is now considered as a specific subset within the broader clinical spectrum of PANS (37, 38). Streptococcal infections have also been suggested to relate to Tourette's Syndrome (TS), a multifactorial and complex disorder that may, in some cases, match the criteria for PANDAS (39). While PANDAS has been proposed as an aetiological subtype of TS (40), the dopamine metabolism pathway is significantly attenuated in this condition, and the relative abundance of *Odoribacter* shows a significant positive correlation with the titer of anti-streptolysin O (37), suggesting that *Odoribacter* may affect the dopamine metabolism pathway and lead to the onset of TD. *Faecalibacterium* can exert anti-inflammatory effects by producing short-chain fatty acids, salicylic acid, and other metabolites. In children showing TD with comorbid ADHD, the abundance of *Faecalibacterium* is low. Studies have shown that changes in dietary structure affect the abundance of *Faecalibacterium*. Excessive intake of food with high monosodium glutamate, caffeine, artificial food dyes, flavorings, fat, sugar, and salt may have a connection with TD (41). In this study, the abundance of *Faecalibacterium*in the TD group increased, which may be related to the differences in the dietary structure of different children.

This study set strict inclusion and exclusion criteria for children with TD, included healthy children as controls, and strictly screened the included healthy children to exclude potential children with TD. The results indicated a decreased abundance of *Bifidobacterium* in the TD group, and the effectiveness of probiotics (Bifidobacterium) in improving TD in patients needs to be further studied. However, this study did not consider issues such as dietary differences, disease duration and a standardized measure of tic symptoms. Moreover, the large-sample studies are still lacking. In further studies, we plan to expand the sample size and stratify tic disorder cases, a questionnaire (including diet structure, lifestyle, health status, medical history, and heredity) will be designed to determine the correlation of TD with intestinal biomarkers and explore the correlation between the pathogenesis of TD and the gut microbiota.

In summary, children with TD showed an abnormal composition of the gut microbiota, suggesting that the microecology of the gut microbiota may have played an important role. In addition, exploration of the changes in the structure and diversity of the gut microbiota can provide clinical evidence for the diagnosis and treatment of children with TD.

## Data Availability Statement

The original contributions presented in the study are publicly available. This data can be found here: https://www-ncbi-nlm-nih-gov.ezproxy.u-pec.fr/Traces/study/?acc=SRP346317&o=acc_s%3Aa.

## Ethics Statement

This study was approved by the Ethics Committee of Wuxi Children's Hospital. Written informed consent to participate in this study was provided by the participants' legal guardian/next of kin.

## Author Contributions

YW, HX, MJ, XH, JW, and YH participated in the design of the study, collected and analyzed the data, and drafted the manuscript. YW, MJ, and XH collected the data. YW, HX, and YH were responsible for analysis, analyzed the data, and contributed to drafting the manuscript. All authors read and approved the final manuscript.

## Funding

This work was supported by the Scientific Research Youth Project of Wuxi Health Committee (Grant No. Q201930).

## Conflict of Interest

The authors declare that the research was conducted in the absence of any commercial or financial relationships that could be construed as a potential conflict of interest.

## Publisher's Note

All claims expressed in this article are solely those of the authors and do not necessarily represent those of their affiliated organizations, or those of the publisher, the editors and the reviewers. Any product that may be evaluated in this article, or claim that may be made by its manufacturer, is not guaranteed or endorsed by the publisher.
